# Neuroimaging correlates of psychological resilience: an Open Science systematic review and meta-analysis

**DOI:** 10.3389/fnimg.2025.1487888

**Published:** 2025-05-13

**Authors:** Allison Kuehn, Maegan L. Calvert, G. Andrew James

**Affiliations:** Department of Psychology, University of Arkansas for Medical Sciences, Little Rock, AR, United States

**Keywords:** psychological resilience, psychiatric disorders, meta-analysis, neuroimaging, amygdala, anterior cingulate

## Abstract

**Introduction:**

While risk factors have been identified for numerous psychiatric disorders, many individuals exposed to these risk factors do not develop psychopathology. A growing neuroimaging literature has sought to find structural and functional brain features that confer psychological resilience against developing psychiatric disorders.

**Methods:**

We conducted a systematic review and meta-analysis of neuroimaging studies associated with psychological resilience. Searches of Pubmed, Embase, Web of Science and PsychInfo yielded 2,658 potentially relevant articles published 2000–2021. Of these, we identified 154 human neuroimaging articles which provided anatomical coordinates of regions promoting resilience against psychiatric disorders including PTSD (44% of articles), schizophrenia (18%), major depressive disorder (14%) and bipolar disorder (12%).

**Results:**

Meta-analysis conducted in GingerALE identified three regions as promoting psychological resilience across disorders (cluster-level FWE *p* < 0.05): left amygdala, right amygdala, and anterior cingulate.

**Discussion:**

We additionally introduce a novel framework for conducting systematic reviews and meta-analyses that is compliant with best practices of Open Science: our publicly viewable systematic review was curated and annotated using the open-source reference manager Zotero, with customizable Python scripts for extracting curated data for meta-analyses. Our methodological pipeline not only permits independent replication of our findings but also supports customization for future neuroimaging meta-analyses.

## Introduction

Numerous environmental and genetic risk factors have been identified for psychiatric disorders including post-traumatic stress disorder (PTSD) (Smoller, 2016; DiGangi et al., 2013; Tortella-Feliu et al., 2019), major depressive disorder (MDD) (Otte et al., 2016; Klengel and Binder, 2013; Lohoff, 2010), bipolar disorder (BD) (Tsuchiya et al., 2003; Alloy et al., 2005; Rowland and Marwaha, 2018), schizophrenia (SZ) (Mäki et al., 2005; McDonald and Murray, 2000; Janoutová et al., 2016), and substance use disorders (SUDs) (Felitti et al., 1998; Sinha and Jastreboff, 2013; Hancock et al., 2018). Yet these risk factors are not deterministic, and many individuals exposed to these risk factors do not develop the associated disorders (Armstrong and Shakespeare-Finch, 2011; Bonanno et al., 2012). These unaffected persons are considered “resilient” and are thought to mount adaptive or compensatory behavioral and brain responses to these risk factors. However, it is unclear if this resilience phenotype (i.e., the state of having risk factors for a psychiatric disorder, but not meeting criteria for a psychiatric diagnosis) is transdiagnostic (buffering against all psychiatric disorders) or disorder-specific. And despite significant neuroimaging progress toward identifying disorder-specific changes in brain structure and function with psychopathology (Suckling and Nestor, 2017; Lawrie et al., 2008; Zhukovsky et al., 2021; Chen et al., 2022; Neurosynth: ptsd; Neurosynth: Addiction; Neurosynth: Schizophrenia; Neurosynth: Major Depressive; Neurosynth: Bipolar Disorder), the neural correlates promoting resilience against psychopathology (if they exist) remain elusive. Characterizing these resilience-enabling responses is a necessary next step for future research seeking to design and implement effective secondary prevention approaches for at-risk persons; e.g., neuromodulation to promote resilience.

A challenge to studying resilience is that this concept has evolved with time and may have different meanings to different investigators (Herrman et al., 2011). As examples, resilience has been variably defined as an outcome following a single traumatic event or following chronic adversity; as an internal personality trait or a product of external support systems; as an innate trait or a learned behavior. Resilience has also been variably described as “protective factors” or “coping behaviors”—although these terms also have broad and potentially ambiguous meanings. In response, the MeSH term “psychological resilience” was formalized in 2009 as “the human ability to adapt in the face of tragedy, trauma, adversity, hardship, and ongoing significant life stressors” (Resilience, Psychological: MeSH Term).

To address this gap in the literature, we conducted a systematic review and meta-analysis of structural and functional brain correlates of psychological resilience. We sought to identify brain regions promoting resilience against developing any psychopathology as well as regions protective against developing specific disorders. We also provide a novel framework for conducting systematic reviews and meta-analyses using non-proprietary software packages (i.e., Zotero reference manager, Python scripts, GingerALE neuroimaging meta-analysis) to promote consistency with best practices of the Open Science Framework.

## Materials and methods

### Literature search

Literature searches were conducted by the UAMS Library's Division of Education & Research Services. Searches were conducted in four abstract databases (PubMed, Embase, Web of Science, PsychInfo) to retrieve studies published from 2000 to 2021 reporting neuroimaging correlates of psychological resilience. Due to this relatively recent introduction of the MeSH term “psychological resilience”, search queries included this MeSH term as well as variants of the word “resilience”. The following search queries were conducted on October 19, 2021:

*PubMed*: #1 (brain mapping[mesh] OR “brain mapping”[tiab] OR “brain region^*^”[tiab] OR neuroimaging[tiab]) AND #2 (resilience, psychological[mesh] OR resiliency[tiab] OR resilience[tiab] OR resilient[tiab]) AND #3 (brain/diagnostic imaging OR fmri OR “functional mri” OR pet[tiab] OR “positron emission tomography” OR fnirs OR “functional near-infrared” OR eeg[tiab] OR electroencephalogra^*^[tiab] OR meg[tiab] OR magnetoencephalography[tiab]) WITH Filters: English, from 01/01/2000 to 10/19/2021.

*Embase*: (“brain mapping”/exp OR “brain mapping” OR “brain region^*^” OR neuroimaging) AND (resilience:ab,ti OR resiliency:ab,ti OR resilient:ab,ti) AND brain:ab,ti AND (“diagnostic imaging”:ab,ti OR fmri:ab,ti OR “functional mri”:ab,ti OR pet:ab,ti OR “positron emission tomography”:ab,ti OR fnirs:ab,ti OR “functional near-infrared”:ab,ti OR eeg:ab,ti OR electroencephalogra^*^:ab,ti OR meg:ab,ti OR magnetoencephalography:ab,ti) AND [2000-2021]/py.

*Web of Science*: “brain mapping” OR “brain region^*^” OR neuroimaging (Topic) and resilience OR resiliency OR resilient (Topic) and fmri OR “functional mri” OR pet OR “positron emission tomography” OR fnirs OR “functional near-infrared” OR eeg OR electroencephalogra^*^ OR meg OR magnetoencephalography (Topic) WITH 2000-01-01 to 2021-10-19 (Publication Date).

*PsychInfo*: S1 (MA brain mapping OR AB brain region^*^ OR AB neuroimaging OR TI brain region^*^ OR TI neuroimaging) AND S2 (MA resilience, psychological OR TI resilien^*^ OR AB resilien^*^) AND [AB (fmri OR “functional MRI” or “positron emission tomography” or fnirs or “functional near-infrared” or EEG OR electroencephalogra^*^ OR MEG OR magnetoencephalogra^*^) OR TI (fmri OR “functional MRI” or “positron emission tomography” or fnirs or “functional near-infrared” or EEG OR electroencephalogra^*^ OR MEG OR magnetoencephalogra^*^)].

**Figure 1:**
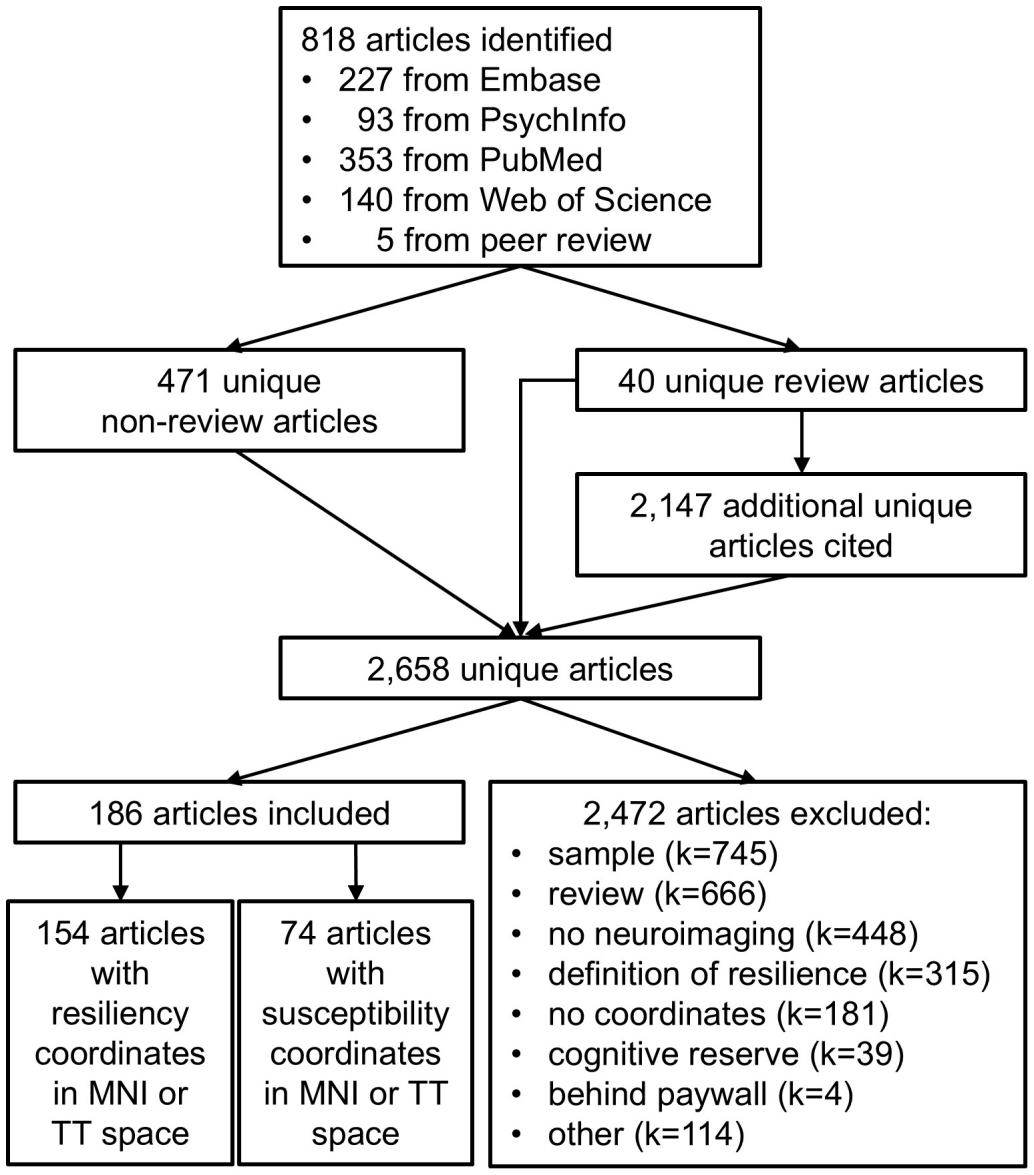
Selection of articles for meta-analysis of psychological resilience. Literature searches of Embase, PsychInfo, PubMed, and Web of Science yielded 818 articles potentially related to psychological resilience. Merging across searches yielded 511 unique articles: 471 unique non-review articles and 40 unique review articles. The review articles cited an additional 2,147 unique articles potentially related to psychological resilience, resulting in a total of 2,658 unique articles. Of these, 2,472 articles were excluded for reasons such as inappropriate sample (*N* = 745), review articles (*N* = 666), no neuroimaging data (*N* = 448), an incorrect definition of resilience (*N* = 315), no neuroimaging coordinates provided (*N* = 181), a definition of resilience focusing on cognitive reserve during healthy aging (*N* = 39), data inaccessible behind paywall (*N* = 4), or other (*N* = 114). A total of 186 articles met inclusion criteria, including 154 articles with coordinates relating to psychological resilience and 74 articles with coordinates relating to susceptibility for psychiatric disorders (to be reviewed in future studies).

Search results are depicted in Figure 1. The searches returned 813 articles (PubMed = 353, Embase = 227, Web of Science = 140, PsychInfo = 93). Five additional articles were identified during peer review for a total of 511 unique articles: 471 unique non-review articles and 40 review articles. Author GAJ reviewed the 471 non-review articles for inclusion or exclusion. GAJ also determined that 23 of the 42 review articles addressed psychological resilience; these 23 articles cited an additional 2,147 unique references, which author AK reviewed for inclusion or exclusion with supervision by GAJ.

### Data curation

Data curation was conducted using the non-proprietary reference manager Zotero to promote open science and reproducibility. Our curated Zotero database is publicly available at https://www.zotero.org/groups/4721296/resilience_systematic_review/library. The Notes section of each article was used to document relevant information for this meta-analysis. Review was conducted in 3 steps or “passes”. Specific inclusion and exclusion criteria at each step are discussed in the next section. In the first pass, author GAJ reviewed the 471 non-review articles for inclusion or exclusion, using the notation “1st keep” and “1st exclude [reason]” to indicate its inclusion or reason for exclusion (Figure 1). For articles meeting multiple exclusion criteria, author GAJ subjectively noted the single most salient reason for exclusion. In the second pass, articles selected for inclusion in the first pass were reviewed for qualitative details about the study including neuroimaging modality, study sample(s), and disease or disorder studied (Figure 1). Articles identified as meeting exclusion criteria during this in-depth second pass were annotated with “2nd exclude [reason]”. The third pass annotated quantitative details from included studies relevant for the meta-analysis. Sample size was annotated as “*N* = X”. Regions identified as promoting resiliency were annotated using a multi-line note. The note's first line “3rd resiliency [MNI/TT]” indicated whether subsequent lines report neuroimaging coordinates using the Montreal Neurological Institute or Talairach-Tourneaux coordinate systems. Subsequent lines in this note indicated the region name and its X, Y, and Z coordinates.

During the data curation process, 74 articles were identified which also provided neuroimaging coordinates associated with susceptibility to psychiatric disorders. While susceptibility was not an *a priori* focus of this manuscript, we notated these coordinates using the annotation “3rd susceptibility [MNI/TT]” for future meta-analysis.

Our literature search yielded 23 relevant review articles which collectively cited 2,147 new unique articles not previously identified by our search. Author AK reviewed the remaining 2,147 articles using the first, second, and third pass criteria described above. These articles were annotated as “4th” pass to distinguish them from articles identified in the “1st pass” primary literature search.^1^

### Inclusion and exclusion criteria

Articles were included in the meta-analysis if they (1) provided neuroimaging coordinates for regions in Talairach-Tourneaux (TT) or Montreal Neurological Institute (MNI) coordinate space (2) in humans (3) promoting psychological resilience and/or susceptibility to psychiatric disorders. Of the 2,658 identified articles, 186 met inclusion criteria. The remaining 2,472 articles were excluded from meta-analysis for: (1) sample, including non-human samples, lacking resilient samples, and case studies (*k* = 745); (2) review articles (*k* = 666); (3) no neuroimaging data (*k* = 448); (4) non-psychological definition of resilience, including resilient computer algorithms and resilience to neurologic injury (*k* = 315); (5) no TT or MNI coordinates provided, including alternate brain atlases and poor EEG source localization (*k* = 181); (6) articles focusing on cognitive reserve, or resilient cognitive function with aging (*k* = 39); (7) inaccessible behind paywall (*k* = 4); and (8) other (*k* = 114). Of the 186 articles that met inclusion criteria, 154 articles provided resilience coordinates and 74 articles provided susceptibility coordinates; note that some articles provided both resilience and susceptibility coordinates.

### Data extraction

The Zotero library was exported as an “Endnote XML” file with “Export Notes” option, which is included as Supplementary material. Custom Python 3.9 scripts imported the resulting.xml file as a ElementTree record tree, traversed this tree to identify records with neuroimaging coordinates for psychological resilience or susceptibility, and wrote those coordinates to format-appropriate text files for neuroimaging meta-analysis via GingerALE (Eickhoff et al., 2009; Turkeltaub et al., 2012; Eickhoff et al., 2012). Disorder-specific neuroimaging coordinates were also saved for the four most common psychiatric disorder subtypes: post-traumatic stress disorder (PTSD), major depressive disorder (MDD), bipolar disorder (BP), and schizophrenia (SZ). Code available via github repository: https://github.com/gandrewjames/resilience_metaanalysis.

### Meta-analyses

Voxelwise activation likelihood estimate (ALE) meta-analytic maps were produced using GingerALE v3.0.2 as follows. First, 3D foci were generated for each study by applying a sample size-dependent Gaussian kernel to study coordinates. During this step, GingerALE converted studies with Talairach coordinates to MNI space as needed. Next, a modeled activation (MA) map was generated by summing voxels' inclusion in 3D foci across studies. Then, a null distribution was generated for thresholding the ALE map by spatially permuting studies' 3D foci. Finally, the MA map was compared against the permuted null distribution at a priori selected statistical thresholds to generate the ALE map (Turkeltaub et al., 2002). Single-group meta-analyses were conducted using the coordinates from all resilience experiments (“ALL”) in GingerALE with the following parameters: output coordinates = MNI, permutations = 1,000, uncorrected *p* = 0.001, and cluster-level FWE *p* = 0.05. GingerALE gave the option of “removing” or “ignoring” experiments with null findings (i.e., no foci); we opted to “ignore” these experiments so that they would still count toward the total number of experiments and thus the meta-analysis permutation statistics would provide a more stringent test. GingerALE also identified foci with coordinates outside of the standard brain mask; we confirmed that all excluded foci were correctly transcribed from source articles (Table 1). This process was repeated for the four most common psychiatric disorders (PTSD, SZ, MDD, BD) to generate disorder-specific resilience ALEs. Note that, since these meta-analyses include data across structural and functional modalities, the meta-analyses can implicate regions relevant to resilience but cannot inform the directionality of these relationships.

**Table 1:** Properties of GingerALE single-group meta-analyses of resilience.

**Meta-analysis**	**Number of experiments**	**Number of null experiments**	**Number of foci**	**Number of foci outside mask**
All disorders	154	14	527	10
PTSD only	66	5	265	7
Schizophrenia only	27	4	89	1
MDD only	21	1	77	1
Bipolar disorder only	18	4	34	0

Contrast meta-analyses were also conducted in GingerALE to assess the specificity of resilience-associated brain regions for psychiatric disorders. Contrasts meta-analyses were conducted as follows. First, a conjunction (ALE) image was generated from the pooled foci coordinates of two disorders (e.g., PTSD and SZ). Next, two ALE contrast images were generated by subtracting each disorder's ALE image from the other (e.g., PSTD-SZ and SZ-PTSD). A null distribution was generated for thresholding the ALE contrast images by randomly assigning the studies' pooled foci into two groupings with the same sizes as each original dataset; this process was repeated for 1,000 permutations to generate the null distribution. Finally, each contrast ALE map was thresholded against this permuted null distribution to find clusters that were significantly more prevalent for one disorder than another (*p*_FDR_ = 0.05). All contrast meta-analyses were conducted with the recommended default settings of 1,000 permutations and *p*_FDR_ = 0.05.

## Results

### Systematic review

The systematic review identified 154 articles or “experiments” reporting regions associated with psychological resilience (Table 1). Fourteen articles yielded null findings (“null experiments”), and the remaining 140 experiments reported 527 regions (“foci”). The four most common disorders evaluated by these articles were PTSD (*k* = 68), schizophrenia (*k* = 27), major depressive disorder (*k* = 21), and bipolar disorder (*k* = 18).

### Meta-analyses

**Figure 2:**
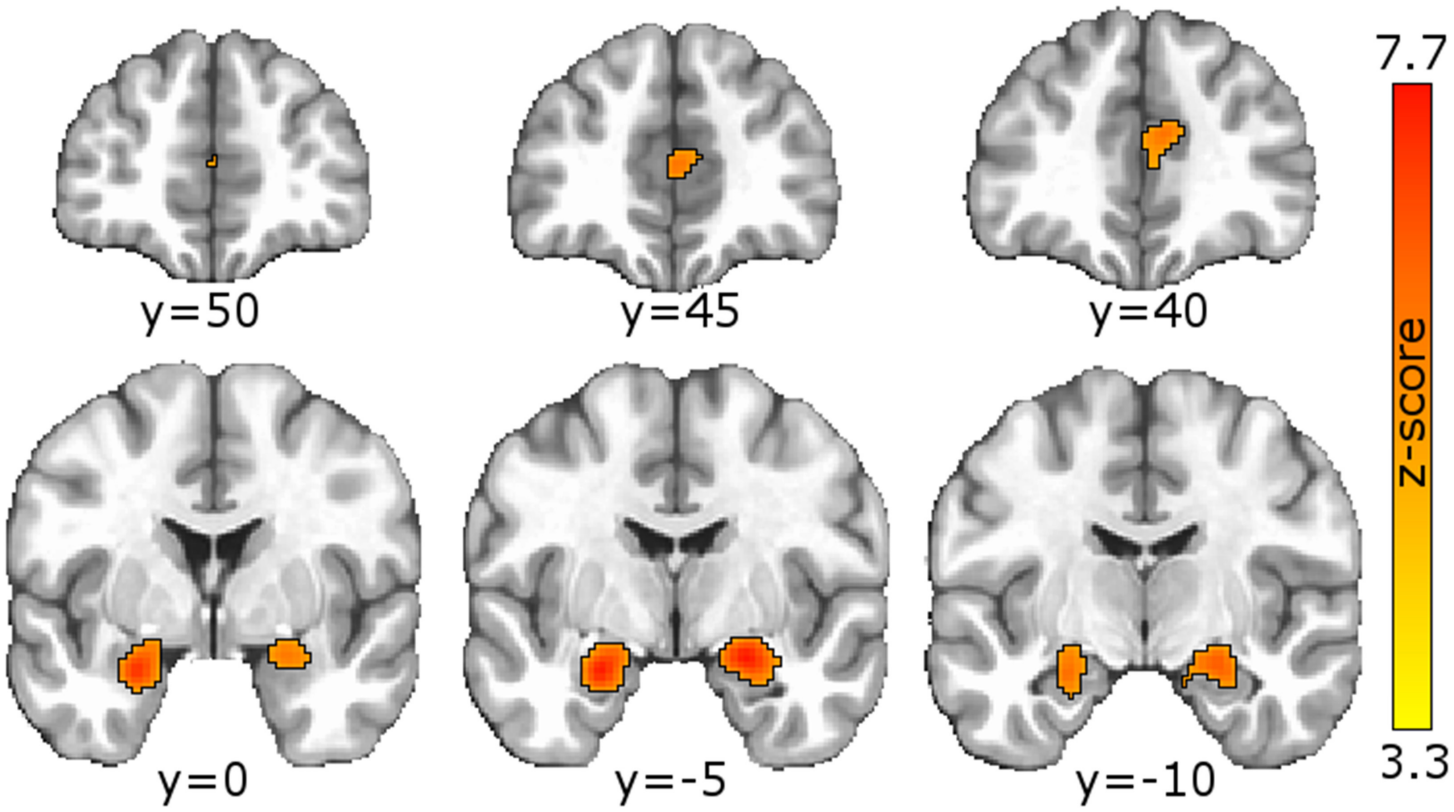
Meta-analysis identified regions associated with psychological resilience across all disorders. GingerALE meta-analysis of the 154 articles generated a modeled activation map of regions relating to psychological resilience. Permutation-based clustering of the modeled activation map (output coordinates = MNI, permutations = 1,000, uncorrected *p* = 0.001, and cluster-level FWE *p* = 0.05) identified 3 regions associated with psychological resilience across all psychiatric disorders: left amygdala (ALE = 0.0566, peak coordinates = −24,−4,−20 MNI; peak *z*-score=7.32), right amygdala (ALE = 0.0613, peak coordinates = 22,−6,−16; peak *z*-score = 7.72); and anterior cingulate (first peak ALE = 0.0319; coordinates = 6, 40, 18; *z-*score = 4.93; second peak ALE = 0.0313, coordinates = 0, 46, 10; *z-*score = 4.85). See Supplementary material for GingerALE meta-analysis of specific disorders and their contrasts, as well as modality-specific meta-analyses.

Single-group meta-analysis of all psychiatric disorders identified 3 regions associated with psychological resilience: left amygdala, right amygdala, and anterior cingulate (Figure 2; Table 2). These three regions appeared in many of the disorder-specific analyses: PTSD resilience was associated with left and right amygdala; schizophrenia resilience was associated with right amygdala and anterior cingulate; and MDD resilience was associated with left amygdala, right amygdala, and anterior cingulate. MDD resilience was additionally associated with left posterior cerebellum. Bipolar disorder resilience was associated with right insula, and is the only disorder for which resilience was not associated with right amygdala, left amygdala, or anterior cingulate.

**Table 2:** Brain regions associated with psychological resilience by disorder.

**Cluster #**	***x***	***y***	***z***	**ALE**	***P***	***Z***	**Label (Nearest Gray Matter within 5 mm)**
**All disorders (*****k** =* **154)**							
1	−24	−4	−20	0.0566	1.28E−13	7.32	Left Cerebrum.Limbic Lobe.Parahippocampal Gyrus.Gray Matter.Amygdala
2	22	−6	−16	0.0613	5.82E−15	7.72	Right Cerebrum.Limbic Lobe.Parahippocampal Gyrus.Gray Matter.Amygdala
3	6	40	18	0.0319	4.15E−07	4.93	Right Cerebrum.Limbic Lobe.Anterior Cingulate.Gray Matter.Brodmann area 32
3	0	46	10	0.0313	6.10E−07	4.85	Left Cerebrum.Limbic Lobe.Anterior Cingulate.Gray Matter.Brodmann area 32
**PTSD (*****k** =* **68)**							
1	−24	−2	−20	0.0312	1.14E−08	5.60	Left Cerebrum.Limbic Lobe.Parahippocampal Gyrus.Gray Matter.Amygdala
1	−26	−14	−20	0.0280	9.42E−08	4.28	Left Cerebrum.Limbic Lobe.Parahippocampal Gyrus.Gray Matter.Hippocampus
2	22	−6	−18	0.0238	1.53E−06	4.68	Right Cerebrum.Limbic Lobe.Parahippocampal Gyrus.Gray Matter.Amygdala
**Schizophrenia (SZ;** ***k** =* **27)**							
1	6	40	18	0.0221	1.71E−07	5.10	Right Cerebrum.Limbic Lobe.Anterior Cingulate.Gray Matter.Brodmann area 32
2	0	16	36	0.0136	7.37E−05	3.80	Left Cerebrum.Limbic Lobe.Cingulate Gyrus.Gray Matter.Brodmann area 32
2	8	20	34	0.0120	1.78E−04	3.57	Right Cerebrum.Frontal Lobe.Cingulate Gyrus.Gray Matter.Brodmann area 32
2	6	20	26	0.0107	3.67E−04	3.38	Right Cerebrum.Limbic Lobe.Cingulate Gyrus.Gray Matter.Brodmann area 24
**Major Depressive Disorder (MDD;** ***k** =* **21)**							
1	−24	−6	−18	0.0264	3.65E−09	5.78	Left Cerebrum.Limbic Lobe.Parahippocampal Gyrus.Gray Matter.Amygdala
2	24	−6	−16	0.0247	1.54E−08	5.54	Right Cerebrum.Limbic Lobe.Parahippocampal Gyrus.Gray Matter.Amygdala
3	−6	18	26	0.0156	1.56E−05	4.16	Left Cerebrum.Limbic Lobe.Cingulate Gyrus.Gray Matter.Brodmann area 24
3	6	14	34	0.0118	1.48E−04	3.62	Right Cerebrum.Limbic Lobe.Cingulate Gyrus.Gray Matter.Brodmann area 24
4	−12	−80	−10	0.0149	2.49E−05	4.06	Left Cerebellum.Posterior Lobe.Declive.Gray Matter
4	−18	−80	−10	0.0149	2.55E−05	4.05	Left Cerebellum.Posterior Lobe.Declive.Gray Matter
**Bipolar Disorder (BD;** ***k** =* **18)**							
1	34	26	−8	0.0316	2.69E−12	6.90	Right Cerebrum.Sub-lobar.Insula.Gray Matter.Brodmann area 13
**PTSD** > **Schizophrenia**							
1	−28	−14	−10	–	0.0035	2.70	Left Cerebrum.Sub-lobar.Lentiform Nucleus.Gray Matter.Putamen
1	−30	−14	−14	–	0.0047	2.60	Left Cerebrum.Limbic Lobe.Parahippocampal Gyrus.Gray Matter.Hippocampus
1	−26	−16	−16	–	0.0048	2.59	Left Cerebrum.Limbic Lobe.Parahippocampal Gyrus.Gray Matter.Hippocampus
**Schizophrenia** > **PTSD**							
Cluster #	x	y	z	ALE	P	Z	Label (Nearest Gray Matter within 5 mm)
1	−3	14	30	–	0.0002	3.54	Left Cerebrum.Limbic Lobe.Cingulate Gyrus.Gray Matter.Brodmann area 24
1	−2	14	34	–	0.0003	3.43	Left Cerebrum.Limbic Lobe.Cingulate Gyrus.Gray Matter.Brodmann area 24
1	7	18	31	–	0.0004	3.35	Right Cerebrum.Limbic Lobe.Cingulate Gyrus.Gray Matter.Brodmann area 24

The contrast meta-analyses of PTSD and schizophrenia were the only contrasts to yield significant results: PTSD resilience was more strongly associated with left hippocampus, while schizophrenia resilience was more strongly associated with anterior cingulate. No other meta-analytic contrasts yielded significant findings.

## Discussion

We present a novel approach for conducting systematic reviews and neuroimaging meta-analyses which embraces the Open Science Framework's best practices for rigor and reproducibility. We demonstrate the advantages for curating this systematic review using the freely-available reference manager Zotero: not only does this approach convey full transparency in articles' selection for inclusion in the meta-analysis, but Zotero allows storage of relevant study data for meta-analyses. We also provide custom Python scripts which allow independent replication of our meta-analysis and can also be modified for other meta-analyses. Our curated systematic review thus conforms to FAIR data principles of Findability, Accessibility, Interoperability, and Reusability. This systematic review was curated during our imaging center's multi-year transition to best practices in Open Science (Bush et al., 2022) and would have further benefitted from other conventions such as pre-registration with PROSPERO (Moher et al., 2014).

Our meta-analysis identified three regions, midline anterior cingulate and bilateral amygdalae, as conferring psychological resilience against psychopathology. Prior meta-analyses have associated anterior cingulate with attentional control (Deng et al., 2018; Freitas et al., 2023; Nee et al., 2007) and amygdalae with affective processing: (Di et al., 2017; García-García et al., 2016; Kirby and Robinson, 2017) two cognitions for which deficits have been reported across diverse psychiatric disorders (Banich et al., 2009; Santens et al., 2020; Hsu et al., 2015; Aldao et al., 2010; Sheppes et al., 2015; Cisler and Olatunji, 2012). While one may be tempted to interpret these findings as evidence that resilience and susceptibility exist along the same continuum (with a “smaller” neuroanatomic feature promoting susceptibility and “larger” one promoting resilience), this meta-analysis by design can neither support nor refute that conclusion. The GingerALE software does not incorporate directionality or magnitude of a neuroanatomic feature, only its presence or absence. An alternative approach would be to encode the magnitude and/or directionality of each feature in the Zotero reference library, adapt our Python code to separately save positive features and negative features in different text files, then conduct a GingerALE contrast meta-analysis of positive and negative features. We intentionally chose to not interpret the source articles' findings beyond regions' neuroanatomic location, as attempting to quantify each region's relationship to resilience as “positive” or “negative” added uncomfortable levels of subjectivity; this was especially true for functional connectivity studies where a “less negative” correlation could be a weakening positive correlation, a strengthening anti-correlation, or a positive correlation becoming anti-correlated. Our goal with these meta-analyses is to qualitatively summarize neuroanatomic contributors to resilience; and through our curation of these meta-analyses via the Open Science Framework, enable future investigators to further explore these regions' modality- or disorder-specific relationships to resilience.

Since 44% of articles in our systematic review (68 of 154) investigated psychological resilience to PTSD, our findings for all disorders could be skewed toward PTSD. Notably, the meta-analysis identified two clusters associated with resilience to PTSD, which had peak coordinates in the left amygdala (−24,−2,−20) and right amygdala (22,−6,−18); these peaks were essentially identical to left and right amygdala peaks reported for all disorders (−24,−4,−20 and 22,−6,−16, respectively). Altered amygdala structure and function has been reported in PTSD (Heim and Nemeroff, 2009), and amygdalae activity among treatment-naïve patients with PTSD during emotion reappraisal (Cisler et al., 2016) and implicit threat processing (Cisler et al., 2015) can predict reductions in PTSD symptoms following trauma-focused cognitive-behavioral therapy (TF-CBT). The association of amygdala with resilience to PTSD is thus not unsurprising. However, several other regions have shown altered structure or function with PTSD including hippocampus, ventromedial prefrontal cortex, and anterior cingulate (Heim and Nemeroff, 2009)—regions our meta-analysis did not associate with resilience to PTSD. Thus, the relationship of neural features to resiliency and susceptibility is likely more complex than a simple dichotomy.

Meta-analyses for schizophrenia, major depressive disorder, and bipolar disorder each had fewer studies than PTSD (respectively, *N* = 27, 21, and 18) and should be interpreted with caution. We report notable overlap between PTSD, schizophrenia, and MDD. Resilience to schizophrenia was associated with rostral anterior cingulate (Brodmann area BA32) whose peak coordinate (Lohoff, 2010; García-García et al., 2016; Suckling and Nestor, 2017) was identical to that reported for all disorders, as well as a dorsal anterior cingulate cluster (BA24) that was also observed in MDD. The amygdalae clusters reported for PTSD were also observed for MDD. Yet MDD resilience was also associated with a left cerebellum cluster not observed for any other disorders, and resilience to bipolar disorder was uniquely associated with right insula.

Only the contrast meta-analyses between PTSD and schizophrenia resilience yielded significant findings. Notably, these contrasts changed considerably with addition of two PTSD studies that were identified during peer review. Prior to peer review, the contrast PTSD > schizophrenia resilience was associated with left amygdala, and the contrast schizophrenia > PTSD resilience identified no regions. After adding these two PTSD studies, the contrast PTSD > schizophrenia resilience shifted from left amygdala to left hippocampus, and the contrast schizophrenia > PTSD resilience became associated with anterior cingulate. We believe that the small number of schizophrenia studies (*k* = 27) could be skewing these findings, and encourage caution when interpreting these meta-analytic contrasts. Similarly, the small study sizes for MDD and BD likely resulted in insufficient power to detect differences among these disorders' neuroanatomic contributors to resilience.

Our meta-analyses merged findings across all neuroimaging modalities to maximize statistical power. We have also conducted resilience meta-analyses for the three most common modalities: task-based fMRI (*k* = 65 experiments), resting-state fMRI (*k* = 34), and structural MRI (*k* = 34). These sub-analyses each show evidence of amygdalae and anterior cingulate associations with resilience across modalities (see Supplementary material). Specifically, task-based fMRI was associated with bilateral amygdalae and right insula, resting-state fMRI was associated with bilateral amygdalae and left middle frontal gyrus, and structural MRI was associated with midline cingulate, left uncus, and left claustrum. Notably, the most prominent features across all modalities (bilateral amygdalae) were observed for two of the three sub-analyses, while the least prominent (anterior cingulate) was only observed for one sub-analysis. An important caveat is that the proportion of disorders represented varies by modality; for example, 32% of structural MRI studies investigated schizophrenia, compared to only 18% of all studies. More resilience studies are needed to fully model the interaction between modality and disorder.

The primary limitation of this work is the relatively recent definition of psychological resilience as a MeSH term in 2009. During peer review, five relevant articles were identified which were not captured by the systematic review because they did not include the terms “psychological resilience” or “resilience”. Our goal is to create a “living” meta-analysis that can be updated annually as new articles are identified, and we encourage authors to contact us with relevant articles which we may have omitted. Consistent with our commitment to Open Science, future code revisions will be posted to github and resulting meta-analyses will be publicly available at OpenNeuro accession number ds0061358 (https://openneuro.org/datasets/ds006138/).

## Data Availability

The original contributions presented in the study are included in the article/Supplementary material. Additionally, the systematic review is available at https://www.zotero.org/groups/4721296/resilience_systematic_review/, accompanying python code is available at https://github.com/gandrewjames/resilience_metaanalysis, and meta-analyses are available at https://openneuro.org/datasets/ds006138/versions.14-APR-2025. Further inquiries can be directed to the corresponding author.

## Footnotes

^1^The 2,147 “4th pass” articles included 626 reviews whose citations were not reviewed for this meta-analysis. However, one 4th pass review article Drevets (1999) was mistakenly annotated as “1st review”, and its 103 citations were added and reviewed. This error was discovered after merging duplicate 1st and 4th pass articles, and thus deleting this article and its citations would also delete articles from the 1st pass literature search. Since none of this review's 103 citations met inclusion criteria, keeping this article's citations marginally inflates the exclusion table but does not impact meta-analysis results.
