# Supplementary material for: Neuroimaging correlates of psychological resilience: an Open Science systematic review and meta-analysis
Source: Front Neuroimaging. 2025 May 13;4:1487888. doi: 10.3389/fnimg.2025.1487888 (PMC12106531; doi:10.3389/fnimg.2025.1487888)
Supplement: Supplementary file 1 [file Data_Sheet_1.zip › Bibliography for Resilience Meta-Analysis.pdf]

Supplemental References

Articles included in the resiliency meta-analysis (1–154) are presented here in the order which they appear in the file "roi_resilience.txt".

1. Leaver AM, Yang H, Siddarth P, Vlasova RM, Krause B, St Cyr N, et al. Resilience and amygdala function in older healthy and depressed adults. J Affect Disord. 2018 Sep;237:27–34.

2. Santarnecchi E, Sprugnoli G, Tatti E, Mencarelli L, Neri F, Momi D, et al. Brain functional connectivity correlates of coping styles. Cogn Affect Behav Neurosci. 2018 Jun;18(3):495–508.

3. Kong F, Ma X, You X, Xiang Y. The resilient brain: psychological resilience mediates the effect of amplitude of low-frequency fluctuations in orbitofrontal cortex on subjective well-being in young healthy adults. Soc Cogn Affect Neurosci. 2018 Sep 4;13(7):755–63.

4. Kong F, Wang X, Hu S, Liu J. Neural correlates of psychological resilience and their relation to life satisfaction in a sample of healthy young adults. Neuroimage. 2015 Dec;123:165–72.

5. Reynaud E, Guedj E, Souville M, Trousselard M, Zendjidjian X, El Khoury-Malhame M, et al. Relationship between emotional experience and resilience: an fMRI study in fire-fighters. Neuropsychologia. 2013 Apr;51(5):845–9.

6. Sinha R, Lacadie CM, Constable RT, Seo D. Dynamic neural activity during stress signals resilient coping. Proc Natl Acad Sci U S A. 2016 Aug 2;113(31):8837–42.

7. van der Werff SJA, Pannekoek JN, Veer IM, van Tol MJ, Aleman A, Veltman DJ, et al. Resilience to childhood maltreatment is associated with increased resting-state functional connectivity of the salience network with the lingual gyrus. Child Abuse Negl. 2013 Nov;37(11):1021–9.

8. Bolwerk A, Mack-Andrick J, Lang FR, Dörfler A, Maihöfner C. How art changes your brain: Differential effects of visual art production and cognitive art evaluation on functional brain connectivity. PLoS ONE [Internet]. 2014;9(7). Available from: https://www.embase.com/search/results?subaction=viewrecord&id=L373444616&from=export

9. Peres JFP, Foerster B, Santana LG, Fereira MD, Nasello AG, Savoia M, et al. Police officers under attack: Resilience implications of an fMRI study. J Psychiatr Res. 2011;45(6):727–34.

10. Singh MK, Chang KD, Kelley RG, Saggar M, Reiss AL, Gotlib IH. Early signs of anomalous neural functional connectivity in healthy offspring of parents with bipolar disorder. Bipolar Disord. 2014;16(7):678–89.

11. Lin T, Vaisvaser S, Fruchter E, Admon R, Wald I, Pine DS, et al. A neurobehavioral account for individual differences in resilience to chronic military stress. Psychol Med. 2015;45(5):1011–23.

12. Wiggins JL, Brotman MA, Adleman NE, Kim P, Wambach CG, Reynolds RC, et al. Neural Markers in Pediatric Bipolar Disorder and Familial Risk for Bipolar Disorder. J Am Acad Child Adolesc Psychiatry. 2017;56(1):67–78.

13. Martz ME, Zucker RA, Schulenberg JE, Heitzeg MM. Psychosocial and neural indicators of resilience among youth with a family history of substance use disorder. Drug Alcohol Depend. 2018;185((Martz M.E., mmartz@umich.edu; Zucker R.A., zuckerra@med.umich.edu; Heitzeg M.M., mheitzeg@med.umich.edu) Department of Psychiatry, University of Michigan, 4250 Plymouth Road, Ann Arbor, MI, United States):198–206.

14. Keynan JN, Cohen A, Jackont G, Green N, Goldway N, Davidov A, et al. Electrical fingerprint of the amygdala guides neurofeedback training for stress resilience. Nat Hum Behav. 2019;3(1):63–73.

15. Guo S, He N, Liu Z, Linli Z, Tao H, Palaniyappan L. Brain-Wide Functional Dysconnectivity in Schizophrenia: Parsing Diathesis, Resilience, and the Effects of Clinical Expression. Can J Psychiatry. 2020;65(1):21–9.

16. Kaldewaij R, Koch SBJ, Hashemi MM, Zhang W, Klumpers F, Roelofs K. Anterior prefrontal brain activity during emotion control predicts resilience to post-traumatic stress symptoms. Nat Hum Behav. 2021;5(8):1055–64.

17. Cisler JM, James GA, Tripathi S, Mletzko T, Heim C, Hu XP, et al. Differential functional connectivity within an emotion regulation neural network among individuals resilient and susceptible to the depressogenic effects of early life stress. Psychological Medicine. 2013 Mar;43(03):507–18.

18. Sepede G, De Berardis D, Campanella D, Perrucci MG, Ferretti A, Salerno RM, et al. Neural correlates of negative emotion processing in bipolar disorder. Prog Neuropsychopharmacol Biol Psychiatry. 2015 Jul 3;60:1–10.

19. Hsu DT, Sanford BJ, Meyers KK, Love TM, Hazlett KE, Wang H, et al. Response of the µ-opioid system to social rejection and acceptance. Mol Psychiatry. 2013 Nov;18(11):1211–7.

20. Wackerhagen C, Veer IM, Erk S, Mohnke S, Lett TA, Wüstenberg T, et al. Amygdala functional connectivity in major depression - disentangling markers of pathology, risk and resilience. Psychol Med. 2020 Dec;50(16):2740–50.

21. Miyagi T, Oishi N, Kobayashi K, Ueno T, Yoshimura S, Murai T, et al. Psychological resilience is correlated with dynamic changes in functional connectivity within the default mode network during a cognitive task. Sci Rep. 2020 Oct 20;10(1):17760.

22. Thorsen AL, de Wit SJ, de Vries FE, Cath DC, Veltman DJ, van der Werf YD, et al. Emotion Regulation in Obsessive-Compulsive Disorder, Unaffected Siblings, and Unrelated Healthy Control Participants. Biol Psychiatry Cogn Neurosci Neuroimaging. 2019 Apr;4(4):352–60.

23. Jeon S, Lee YJ, Park I, Kim N, Kim S, Jun JY, et al. Resting State Functional Connectivity of the Thalamus in North Korean Refugees with and without Posttraumatic Stress Disorder. Sci Rep. 2020 Feb 21;10(1):3194.

24. Nord C, Halahakoon D, Lally N, Limbachya T, Pilling S, Rosier R. The neural basis of hot and cold cognition in depressed patients, unaffected relatives, and low-risk healthy controls: An fMRI investigation. J Affect Disord. 2020 Sep 1;274:389–98.

25. Hanson JL, Gillmore AD, Yu T, Holmes CJ, Hallowell ES, Barton AW, et al. A Family Focused Intervention Influences Hippocampal-Prefrontal Connectivity Through Gains in Self-Regulation. Child Dev. 2019 Jul;90(4):1389–401.

26. Fujisawa TX, Jung M, Kojima M, Saito DN, Kosaka H, Tomoda A. Neural Basis of Psychological Growth following Adverse Experiences: A Resting-State Functional MRI Study. PLoS One. 2015;10(8):e0136427.

27. Meng L, Chen Y, Xu X, Chen T, Lui S, Huang X, et al. The neurobiology of brain recovery from traumatic stress: A longitudinal DTI study. J Affect Disord. 2018 Jan 1;225:577–84.

28. Eckstrand KL, Hanford LC, Bertocci MA, Chase HW, Greenberg T, Lockovich J, et al. Trauma-associated anterior cingulate connectivity during reward learning predicts affective and anxiety states in young adults. Psychol Med. 2019 Aug;49(11):1831–40.

29. Amico F, Meisenzahl E, Koutsouleris N, Reiser M, Möller HJ, Frodl T. Structural MRI correlates for vulnerability and resilience to major depressive disorder. J Psychiatry Neurosci. 2011 Jan;36(1):15–22.

30. Uchida M, Biederman J, Gabrieli JDE, Micco J, de Los Angeles C, Brown A, et al. Emotion regulation ability varies in relation to intrinsic functional brain architecture. Soc Cogn Affect Neurosci. 2015 Dec;10(12):1738–48.

31. Martz ME, Cope LM, Hardee JE, Brislin SJ, Weigard A, Zucker RA, et al. Frontostriatal Resting State Functional Connectivity in Resilient and Non-Resilient Adolescents with a Family History of Alcohol Use Disorder. J Child Adolesc Psychopharmacol. 2019 Aug;29(7):508–15.

32. Singh MK, Leslie SM, Packer MM, Weisman EF, Gotlib IH. Limbic Intrinsic Connectivity in Depressed and High-Risk Youth. J Am Acad Child Adolesc Psychiatry. 2018 Oct;57(10):775-785.e3.

33. Kilpatrick LA, Istrin JJ, Gupta A, Naliboff BD, Tillisch K, Labus JS, et al. Sex commonalities and differences in the relationship between resilient personality and the intrinsic connectivity of the salience and default mode networks. Biol Psychol. 2015 Dec;112:107–15.

34. Hirshfeld-Becker DR, Gabrieli JDE, Shapero BG, Biederman J, Whitfield-Gabrieli S, Chai XJ. Intrinsic Functional Brain Connectivity Predicts Onset of Major Depression Disorder in Adolescence: A Pilot Study. Brain Connect. 2019 Jun;9(5):388–98.

35. Shi L, Ren Z, Qiu J. High Thought Control Ability, High Resilience: The Effect of Temporal Cortex and Insula Connectivity. Neuroscience. 2021 Sep 15;472:60–7.

36. Dolcos S, Hu Y, Iordan AD, Moore M, Dolcos F. Optimism and the brain: trait optimism mediates the protective role of the orbitofrontal cortex gray matter volume against anxiety. Soc Cogn Affect Neurosci. 2016 Feb;11(2):263–71.

37. Miller GE, Chen E, Armstrong CC, Carroll AL, Ozturk S, Rydland KJ, et al. Functional connectivity in central executive network protects youth against cardiometabolic risks linked with neighborhood violence. Proc Natl Acad Sci U S A. 2018 Nov 20;115(47):12063–8.

38. Grueschow M, Stenz N, Thörn H, Ehlert U, Breckwoldt J, Brodmann Maeder M, et al. Real-world stress resilience is associated with the responsivity of the locus coeruleus. Nat Commun. 2021 Apr 15;12(1):2275.

39. Charpentier CJ, De Martino B, Sim AL, Sharot T, Roiser JP. Emotion-induced loss aversion and striatal-amygdala coupling in low-anxious individuals. Soc Cogn Affect Neurosci. 2016 Apr;11(4):569–79.

40. Kennis M, Rademaker AR, van Rooij SJH, Kahn RS, Geuze E. Resting state functional connectivity of the anterior cingulate cortex in veterans with and without post-traumatic stress disorder. Hum Brain Mapp. 2015 Jan;36(1):99–109.

41. Morein-Zamir S, Simon Jones P, Bullmore ET, Robbins TW, Ersche KD. Prefrontal hypoactivity associated with impaired inhibition in stimulant-dependent individuals but evidence for hyperactivation in their unaffected siblings. Neuropsychopharmacology. 2013 Sep;38(10):1945–53.

42. Macoveanu J, Miskowiak K, Kessing LV, Vinberg M, Siebner HR. Healthy co-twins of patients with affective disorders show reduced risk-related activation of the insula during a monetary gambling task. J Psychiatry Neurosci. 2016 Jan;41(1):38–47.

43. Zhou Z, Zhu G, Hariri AR, Enoch MA, Scott D, Sinha R, et al. Genetic variation in human NPY expression affects stress response and emotion. Nature. 2008 Apr 24;452(7190):997–1001.

44. Waugh CE, Wager TD, Fredrickson BL, Noll DC, Taylor SF. The neural correlates of trait resilience when anticipating and recovering from threat. Soc Cogn Affect Neurosci. 2008 Dec;3(4):322–32.

45. Schweizer S, Walsh ND, Stretton J, Dunn VJ, Goodyer IM, Dalgleish T. Enhanced emotion regulation capacity and its neural substrates in those exposed to moderate childhood adversity. Soc Cogn Affect Neurosci. 2016 Feb;11(2):272–81.

46. Miller GE, Chen E, Finegood ED, Lam PH, Weissman-Tsukamoto R, Leigh AKK, et al. Resting-State Functional Connectivity of the Central Executive Network Moderates the Relationship Between Neighborhood Violence and Proinflammatory Phenotype in Children. Biol Psychiatry. 2021 Aug 1;90(3):165–72.

47. Speer ME, Bhanji JP, Delgado MR. Savoring the past: positive memories evoke value representations in the striatum. Neuron. 2014 Nov 19;84(4):847–56.

48. Hyde LW, Gorka A, Manuck SB, Hariri AR. Perceived social support moderates the link between threat-related amygdala reactivity and trait anxiety. Neuropsychologia. 2011 Mar;49(4):651–6.

49. Stevens JS, Harnett NG, Lebois LAM, van Rooij SJH, Ely TD, Roeckner A, et al. Brain-Based Biotypes of Psychiatric Vulnerability in the Acute Aftermath of Trauma. Am J Psychiatry. 2021 Oct 14;appiajp202120101526.

50. Zhang S, Cui J, Zhang Z, Wang Y, Liu R, Chen X, et al. Functional connectivity of amygdala subregions predicts vulnerability to depression following the COVID-19 pandemic. J Affect Disord. 2021 Oct 1;

51. Ganzel BL, Kim P, Glover GH, Temple E. Resilience after 9/11: multimodal neuroimaging evidence for stress-related change in the healthy adult brain. Neuroimage. 2008 Apr 1;40(2):788–95.

52. Loth E, Poline JB, Thyreau B, Jia T, Tao C, Lourdusamy A, et al. Oxytocin receptor genotype modulates ventral striatal activity to social cues and response to stressful life events. Biol Psychiatry. 2014 Sep 1;76(5):367–76.

53. Grant MM, Wood K, Sreenivasan K, Wheelock M, White D, Thomas J, et al. Influence of early life stress on intra- and extra-amygdaloid causal connectivity. Neuropsychopharmacology. 2015 Jun;40(7):1782–93.

54. Heitzeg MM, Cope LM, Martz ME, Hardee JE, Zucker RA. Brain activation to negative stimuli mediates a relationship between adolescent marijuana use and later emotional functioning. Dev Cogn Neurosci. 2015 Dec;16:71–83.

55. Demers LA, McKenzie KJ, Hunt RH, Cicchetti D, Cowell RA, Rogosch FA, et al. Separable Effects of Childhood Maltreatment and Adult Adaptive Functioning on Amygdala Connectivity During Emotion Processing. Biol Psychiatry Cogn Neurosci Neuroimaging. 2018 Feb;3(2):116–24.

56. Williams B, Jalilianhasanpour R, Matin N, Fricchione GL, Sepulcre J, Keshavan MS, et al. Individual differences in corticolimbic structural profiles linked to insecure attachment and coping styles in motor functional neurological disorders. J Psychiatr Res. 2018 Jul;102:230–7.

57. Gatt J, Burton K, Routledge K, Grasby K, Korgaonkar M, Grieve S, et al. A negative association between brainstem pontine grey-matter volume, well-being and resilience in healthy twins. JOURNAL OF PSYCHIATRY & NEUROSCIENCE. 2018 Nov;43(6):386–95.

58. Mirman A, Bick A, Kalla C, Canetti L, Segman R, Dan R, et al. The imprint of childhood adversity on emotional processing in high functioning young adults. HUMAN BRAIN MAPPING. 2021 Feb 15;42(3):615–25.

59. Britton J, Phan K, Taylor S, Fig L, Liberzon I. Corticolimbic blood flow in posttraumatic stress disorder during script-driven imagery. BIOLOGICAL PSYCHIATRY. 2005 Apr 15;57(8):832–40.

60. Dolcos S, Hu Y, Williams C, Bogdan PC, Hohl K, Berenbaum H, et al. Cultivating Affective Resilience: Proof-of-Principle Evidence of Translational Benefits From a Novel Cognitive-Emotional Training Intervention. Front Psychol. 2021;12:585536.

61. Herremans SC, De Raedt R, Van Schuerbeek P, Marinazzo D, Matthys F, De Mey J, et al. Accelerated HF-rTMS Protocol has a Rate-Dependent Effect on dACC Activation in Alcohol-Dependent Patients: An Open-Label Feasibility Study. Alcohol Clin Exp Res. 2016;40(1):196–205.

62. Iadipaolo AS, Marusak HA, Sala-Hamrick K, Crespo LM, Thomason ME, Rabinak CA. Behavioral activation sensitivity and default mode network-subgenual cingulate cortex connectivity in youth. Behav Brain Res. 2017 Aug 30;333:135–41.

63. Kaldewaij R, Koch SBJ, Zhang W, Hashemi MM, Klumpers F, Roelofs K. Frontal Control Over Automatic Emotional Action Tendencies Predicts Acute Stress Responsivity. Biol Psychiatry Cogn Neurosci Neuroimaging. 2019 Nov;4(11):975–83.

64. Nelson EC, Agrawal A, Heath AC, Bogdan R, Sherva R, Zhang B, et al. Evidence of CNIH3 involvement in opioid dependence. Mol Psychiatry. 2016 May;21(5):608–14.

65. Nørgaard M, Ganz M, Svarer C, Fisher PM, Churchill NW, Beliveau V, et al. Brain Networks Implicated in Seasonal Affective Disorder: A Neuroimaging PET Study of the Serotonin Transporter. Front Neurosci. 2017;11:614.

66. Underwood R, Mason L, O'Daly O, Dalton J, Simmons A, Barker GJ, et al. You read my mind: fMRI markers of threatening appraisals in people with persistent psychotic experiences. NPJ Schizophr [Internet]. 2021;7(1). Available from: https://www.embase.com/search/results?subaction=viewrecord&id=L2013919590&from=export

67. Falconer E, Bryant R, Felmingham K, Kemp A, Olivieri G, Peduto A, et al. The neural networks of inhibitory control in post-traumatic stress disorder. Acta Neuropsychiatr. 2006 Dec;18(6):323–323.

68. Bremner JD, Vermetten E, Vythilingam M, Afzal N, Schmahl C, Elzinga B, et al. Neural correlates of the classic color and emotional stroop in women with abuse-related posttraumatic stress disorder. Biological Psychiatry. 2004 Mar 15;55(6):612–20.

69. Morey RA, Petty CM, Cooper DA, LaBar KS, McCarthy G. Neural systems for executive and emotional processing are modulated by symptoms of posttraumatic stress disorder in Iraq War veterans. Psychiatry Research: Neuroimaging. 2008 Jan 15;162(1):59–72.

70. Eckart C, Stoppel C, Kaufmann J, Tempelmann C, Hinrichs H, Elbert T, et al. Structural alterations in lateral prefrontal, parietal and posterior midline regions of men with chronic posttraumatic stress disorder. Journal of Psychiatry and Neuroscience. 2011 May 1;36(3):176–86.

71. Chen S, Xia W, Li L, Liu J, He Z, Zhang Z, et al. Gray matter density reduction in the insula in fire survivors with posttraumatic stress disorder: A voxel-based morphometric study. Psychiatry Research: Neuroimaging. 2006 Jan 30;146(1):65–72.

72. Brown VM, LaBar KS, Haswell CC, Gold AL, McCarthy G, Morey RA. Altered Resting-State Functional Connectivity of Basolateral and Centromedial Amygdala Complexes in Posttraumatic Stress Disorder. Neuropsychopharmacol. 2014 Jan;39(2):351–9.

73. Zhang X, Zhang J, Wang L, Li R, Zhang W. Altered resting-state functional connectivity of the amygdala in Chinese earthquake survivors. Progress in Neuro-Psychopharmacology and Biological Psychiatry. 2016 Feb 4;65:208–14.

74. Sripada RK, King AP, Garfinkel SN, Wang X, Sripada CS, Welsh RC, et al. Altered resting-state amygdala functional connectivity in men with posttraumatic stress disorder. Journal of Psychiatry and Neuroscience. 2012 Jul 1;37(4):241–9.

75. Stevens JS, Ely TD, Sawamura T, Guzman D, Bradley B, Ressler KJ, et al. Childhood Maltreatment Predicts Reduced Inhibition-Related Activity in the Rostral Anterior Cingulate in Ptsd, but Not Trauma-Exposed Controls. Depression and Anxiety. 2016;33(7):614–22.

76. Sripada RK, King AP, Welsh RC, Garfinkel SN, Wang X, Sripada CS, et al. Neural Dysregulation in Posttraumatic Stress Disorder: Evidence for Disrupted Equilibrium Between Salience and Default Mode Brain Networks. Psychosomatic Medicine. 2012 Dec;74(9):904–11.

77. Shin LM, Wright CI, Cannistraro PA, Wedig MM, McMullin K, Martis B, et al. A Functional Magnetic Resonance Imaging Study of Amygdala and Medial Prefrontal Cortex Responses to Overtly Presented Fearful Faces in Posttraumatic Stress Disorder. Archives of General Psychiatry. 2005 Mar 1;62(3):273–81.

78. Rogers MA, Yamasue H, Abe O, Yamada H, Ohtani T, Iwanami A, et al. Smaller amygdala volume and reduced anterior cingulate gray matter density associated with history of post-traumatic stress disorder. Psychiatry Research: Neuroimaging. 2009 Dec 30;174(3):210–6.

79. New AS, Fan J, Murrough JW, Liu X, Liebman RE, Guise KG, et al. A Functional Magnetic Resonance Imaging Study of Deliberate Emotion Regulation in Resilience and Posttraumatic Stress Disorder. Biological Psychiatry. 2009 Oct 1;66(7):656–64.

80. Morey RA, Haswell CC, Hooper SR, De Bellis MD. Amygdala, Hippocampus, and Ventral Medial Prefrontal Cortex Volumes Differ in Maltreated Youth with and without Chronic Posttraumatic Stress Disorder. Neuropsychopharmacology. 2016 Feb;41(3):791–801.

81. Kasai K, Yamasue H, Gilbertson MW, Shenton ME, Rauch SL, Pitman RK. Evidence for Acquired Pregenual Anterior Cingulate Gray Matter Loss from A Twin Study of Combat-Related Post-Traumatic Stress Disorder. Biol Psychiatry. 2008 Mar 15;63(6):550–6.

82. Freeman T, Kimbrell T, Booe L, Myers M, Cardwell D, Lindquist DM, et al. Evidence of resilience: Neuroimaging in former prisoners of war. Psychiatry Research: Neuroimaging. 2006 Jan 30;146(1):59–64.

83. Felmingham K, Williams LM, Whitford TJ, Falconer E, Kemp AH, Peduto A, et al. Duration of posttraumatic stress disorder predicts hippocampal grey matter loss. NeuroReport. 2009 Oct 28;20(16):1402–6.

84. Tseng WL, Bones BL, Kayser RR, Olsavsky AK, Fromm SJ, Pine DS, et al. An fMRI study of emotional face encoding in youth at risk for bipolar disorder. Eur psychiatr. 2015 Jan;30(1):94–8.

85. Sambataro F, Mattay VS, Thurin K, Safrin M, Rasetti R, Blasi G, et al. Altered Cerebral Response During Cognitive Control: A Potential Indicator of Genetic Liability for Schizophrenia. Neuropsychopharmacology. 2013 Apr;38(5):846–53.

86. Pompei F, Jogia J, Tatarelli R, Girardi P, Rubia K, Kumari V, et al. Familial and disease specific abnormalities in the neural correlates of the Stroop Task in Bipolar Disorder. NeuroImage. 2011 Jun 1;56(3):1677–84.

87. Linke J, King AV, Rietschel M, Strohmaier J, Hennerici M, Gass A, et al. Increased Medial Orbitofrontal and Amygdala Activation: Evidence for a Systems-Level Endophenotype of Bipolar I Disorder. AJP. 2012 Mar;169(3):316–25.

88. Kempton MJ, Haldane M, Jogia J, Grasby PM, Collier D, Frangou S. Dissociable Brain Structural Changes Associated with Predisposition, Resilience, and Disease Expression in Bipolar Disorder. J Neurosci. 2009 Sep 2;29(35):10863–8.

89. Burt KB, Whelan R, Conrod PJ, Banaschewski T, Barker GJ, Bokde ALW, et al. Structural brain correlates of adolescent resilience. Journal of Child Psychology and Psychiatry. 2016;57(11):1287–96.

90. van Amelsvoort T, Daly E, Henry J, Robertson D, Ng V, Owen M, et al. Brain Anatomy in Adults With Velocardiofacial Syndrome With and WithoutSchizophrenia: Preliminary Results of a Structural Magnetic Resonance Imaging Study. Archives of General Psychiatry. 2004 Nov 1;61(11):1085–96.

91. Anticevic A, Haut K, Murray JD, Repovs G, Yang GJ, Diehl C, et al. Association of Thalamic Dysconnectivity and Conversion to Psychosis in Youth and Young Adults at Elevated Clinical Risk. JAMA Psychiatry. 2015 Sep 1;72(9):882–91.

92. Chow EWC, Ho A, Wei C, Voormolen EHJ, Crawley AP, Bassett AS. Association of Schizophrenia in 22q11.2 Deletion Syndrome and Gray Matter Volumetric Deficits in the Superior Temporal Gyrus. AJP. 2011 May;168(5):522–9.

93. Milad MR, Pitman RK, Ellis CB, Gold AL, Shin LM, Lasko NB, et al. Neurobiological Basis of Failure to Recall Extinction Memory in Posttraumatic Stress Disorder. Biol Psychiatry. 2009 Dec 15;66(12):1075–82.

94. Koch SBJ, Zuiden M van, Nawijn L, Frijling JL, Veltman DJ, Olff M. Decreased uncinate fasciculus tract integrity in male and female patients with PTSD: a diffusion tensor imaging study. Journal of Psychiatry and Neuroscience. 2017 Sep 1;42(5):331–42.

95. Koch SB, van Zuiden M, Nawijn L, Frijling JL, Veltman DJ, Olff M. Intranasal Oxytocin Administration Dampens Amygdala Reactivity towards Emotional Faces in Male and Female PTSD Patients. Neuropsychopharmacology. 2016 May;41(6):1495–504.

96. Helpman L, Marin MF, Papini S, Zhu X, Sullivan GM, Schneier F, et al. Neural changes in extinction recall following prolonged exposure treatment for PTSD: A longitudinal fMRI study. Neuroimage Clin. 2016 Oct 10;12:715–23.

97. Roberts G, Lord A, Frankland A, Wright A, Lau P, Levy F, et al. Functional Dysconnection of the Inferior Frontal Gyrus in Young People With Bipolar Disorder or at Genetic High Risk. Biological Psychiatry. 2017 Apr 15;81(8):718–27.

98. Pompei F, Dima D, Rubia K, Kumari V, Frangou S. Dissociable functional connectivity changes during the Stroop task relating to risk, resilience and disease expression in bipolar disorder. NeuroImage. 2011 Jul 15;57(2):576–82.

99. McIntosh AM, Job DE, Moorhead TWJ, Harrison LK, Forrester K, Lawrie SM, et al. Voxel-based morphometry of patients with schizophrenia or bipolar disorder and their unaffected relatives. Biological Psychiatry. 2004 Oct 15;56(8):544–52.

100. Lui S, Yao L, Xiao Y, Keedy SK, Reilly JL, Keefe RS, et al. Resting-state brain function in schizophrenia and psychotic bipolar probands and their first-degree relatives. Psychol Med. 2015 Jan;45(1):97–108.

101. Khadka S, Meda SA, Stevens MC, Glahn DC, Calhoun VD, Sweeney JA, et al. Is Aberrant Functional Connectivity A Psychosis Endophenotype? A Resting State Functional Magnetic Resonance Imaging Study. Biol Psychiatry. 2013 Sep 15;74(6):458–66.

102. Frangou S, Dima D, Jogia J. Towards person-centered neuroimaging markers for resilience and vulnerability in Bipolar Disorder. Neuroimage. 2017 Jan 15;145(Pt B):230–7.

103. Frangou S. Brain structural and functional correlates of resilience to Bipolar Disorder. Front Hum Neurosci. 2012 Jan 27;5:184.

104. Fischer AS, Camacho MC, Ho TC, Whitfield-Gabrieli S, Gotlib IH. Neural Markers of Resilience in Adolescent Females at Familial Risk for Major Depressive Disorder. JAMA Psychiatry. 2018 May;75(5):493–502.

105. Dima D, Roberts RE, Frangou S. Connectomic markers of disease expression, genetic risk and resilience in bipolar disorder. Transl Psychiatry. 2016 Jan;6(1):e706.

106. Rougemont-Bücking A, Linnman C, Zeffiro TA, Zeidan MA, Lebron-Milad K, Rodriguez-Romaguera J, et al. Altered Processing of Contextual Information during Fear Extinction in PTSD: An fMRI Study. CNS Neurosci Ther. 2010 Apr 16;17(4):227–36.

107. Bryant RA, Felmingham K, Whitford TJ, Kemp A, Hughes G, Peduto A, et al. Rostral anterior cingulate volume predicts treatment response to cognitive-behavioural therapy for posttraumatic stress disorder. J Psychiatry Neurosci. 2008 Mar;33(2):142–6.

108. Dennison MJ, Sheridan MA, Busso DS, Jenness JL, Peverill M, Rosen ML, et al. Neurobehavioral markers of resilience to depression amongst adolescents exposed to child abuse. J Abnorm Psychol. 2016 Nov;125(8):1201–12.

109. Zhang J, Tan Q, Yin H, Zhang X, Huan Y, Tang L, et al. Decreased gray matter volume in the left hippocampus and bilateral calcarine cortex in coal mine flood disaster survivors with recent onset PTSD. Psychiatry Research: Neuroimaging. 2011 May 31;192(2):84–90.

110. van Rooij SJH, Kennis M, Vink M, Geuze E. Predicting Treatment Outcome in PTSD: A Longitudinal Functional MRI Study on Trauma-Unrelated Emotional Processing. Neuropsychopharmacol. 2016 Mar;41(4):1156–65.

111. Sullivan DR, Morrison FG, Wolf EJ, Logue MW, Fortier CB, Salat DH, et al. The PPM1F gene moderates the association between PTSD and cortical thickness. J Affect Disord. 2019 Dec 1;259:201–9.

112. Qi S, Mu Y, Liu K, Zhang J, Huan Y, Tan Q, et al. Cortical inhibition deficits in recent onset PTSD after a single prolonged trauma exposure. NeuroImage: Clinical. 2013 Jan 1;3:226–33.

113. O'Doherty DCM, Tickell A, Ryder W, Chan C, Hermens DF, Bennett MR, et al. Frontal and subcortical grey matter reductions in PTSD. Psychiatry Research: Neuroimaging. 2017 Aug 30;266:1–9.

114. Misaki M, Phillips R, Zotev V, Wong CK, Wurfel BE, Krueger F, et al. Connectome-wide investigation of altered resting-state functional connectivity in war veterans with and without posttraumatic stress disorder. Neuroimage Clin. 2017 Oct 31;17:285–96.

115. Liu Y, Li B, Feng N, Pu H, Zhang X, Lu H, et al. Perfusion Deficits and Functional Connectivity Alterations in Memory-Related Regions of Patients with Post-Traumatic Stress Disorder. PLOS ONE. 2016 May 23;11(5):e0156016.

116. Liu Y, Li YJ, Luo EP, Lu HB, Yin H. Cortical Thinning in Patients with Recent Onset Post-Traumatic Stress Disorder after a Single Prolonged Trauma Exposure. PLOS ONE. 2012 Jun 13;7(6):e39025.

117. Herringa R, Phillips M, Almeida J, Insana S, Germain A. Post-traumatic stress symptoms correlate with smaller subgenual cingulate, caudate, and insula volumes in unmedicated combat veterans. Psychiatry Research: Neuroimaging. 2012 Aug 1;203(2):139–45.

118. Blair KS, Vythilingam M, Crowe SL, McCaffrey DE, Ng P, Wu CC, et al. Cognitive control of attention is differentially affected in trauma-exposed individuals with and without post-traumatic stress disorder. Psychological Medicine. 2013 Jan;43(1):85–95.

119. Sarıçiçek A, Yalın N, Hıdıroğlu C, Çavuşoğlu B, Taş C, Ceylan D, et al. Neuroanatomical correlates of genetic risk for bipolar disorder: A voxel-based morphometry study in bipolar type I patients and healthy first degree relatives. Journal of Affective Disorders. 2015 Nov 1;186:110–8.

120. Nery FG, Gigante AD, Amaral JA, Fernandes FBF, Berutti M, Almeida KM, et al. Gray matter volumes in patients with bipolar disorder and their first-degree relatives. Psychiatry Research: Neuroimaging. 2015 Nov 30;234(2):188–93.

121. Kanske P, Schönfelder S, Forneck J, Wessa M. Impaired regulation of emotion: neural correlates of reappraisal and distraction in bipolar disorder and unaffected relatives. Transl Psychiatry. 2015 Jan;5(1):e497–e497.

122. Eker C, Simsek F, Yılmazer EE, Kitis O, Cinar C, Eker OD, et al. Brain regions associated with risk and resistance for bipolar I disorder: a voxel-based MRI study of patients with bipolar disorder and their healthy siblings. Bipolar Disorders. 2014;16(3):249–61.

123. Chaddock CA, Barker GJ, Marshall N, Schulze K, Hall MH, Fern A, et al. White matter microstructural impairments and genetic liability to familial bipolar I disorder. The British Journal of Psychiatry. 2009 Jun;194(6):527–34.

124. Broome MR, Fusar-Poli P, Matthiasson P, Woolley JB, Valmaggia L, Johns LC, et al. Neural correlates of visuospatial working memory in the 'at-risk mental state.' Psychological Medicine. 2010 Dec;40(12):1987–99.

125. Broome MR, Matthiasson P, Fusar-Poli P, Woolley JB, Johns LC, Tabraham P, et al. Neural correlates of executive function and working memory in the 'at-risk mental state.' The British Journal of Psychiatry. 2009 Jan;194(1):25–33.

126. Borgwardt SJ, Picchioni MM, Ettinger U, Toulopoulou T, Murray R, McGuire PK. Regional Gray Matter Volume in Monozygotic Twins Concordant and Discordant for Schizophrenia. Biological Psychiatry. 2010 May 15;67(10):956–64.

127. Borgwardt SJ, McGuire PK, Aston J, Gschwandtner U, Pflüger MO, Stieglitz RD, et al. Reductions in frontal, temporal and parietal volume associated with the onset of psychosis. Schizophrenia Research. 2008 Dec 1;106(2):108–14.

128. Borgwardt SJ, Riecher-Rössler A, Dazzan P, Chitnis X, Aston J, Drewe M, et al. Regional Gray Matter Volume Abnormalities in the At Risk Mental State. Biological Psychiatry. 2007 May 15;61(10):1148–56.

129. Boos HBM, Cahn W, van Haren NEM, Derks EM, Brouwer RM, Schnack HG, et al. Focal And Global Brain Measurements in Siblings of Patients With Schizophrenia. Schizophr Bull. 2012 Jul;38(4):814–25.

130. Allen P, Stephan KE, Mechelli A, Day F, Ward N, Dalton J, et al. Cingulate activity and fronto-temporal connectivity in people with prodromal signs of psychosis. Neuroimage. 2010 Jan 1;49(1–3):947–55.

131. Witthaus H, Brüne M, Kaufmann C, Bohner G, Özgürdal S, Gudlowski Y, et al. White matter abnormalities in subjects at ultra high-risk for schizophrenia and first-episode schizophrenic patients. Schizophrenia Research. 2008 Jul 1;102(1):141–9.

132. Walterfang M, McGuire PK, Yung AR, Phillips LJ, Velakoulis D, Wood SJ, et al. White matter volume changes in people who develop psychosis. The British Journal of Psychiatry. 2008 Sep;193(3):210–5.

133. Spence SA, Liddle PF, Stefan MD, Hellewell JSE, Sharma T, Friston KJ, et al. Functional anatomy of verbal fluency in people with schizophrenia and those at genetic risk: Focal dysfunction and distributed disconnectivity reappraised. The British Journal of Psychiatry. 2000 Jan;176(1):52–60.

134. Smieskova R, Fusar-Poli P, Aston J, Simon A, Bendfeldt K, Lenz C, et al. Insular volume abnormalities associated with different transition probabilities to psychosis. Psychol Med. 2012 Aug;42(8):1613–25.

135. Mechelli A, Riecher-Rössler A, Meisenzahl EM, Tognin S, Wood SJ, Borgwardt SJ, et al. Neuroanatomical Abnormalities That Predate the Onset of Psychosis: A Multicenter Study. Archives of General Psychiatry. 2011 May 2;68(5):489–95.

136. Marjoram D, Job DE, Whalley HC, Gountouna VE, McIntosh AM, Simonotto E, et al. A visual joke fMRI investigation into Theory of Mind and enhanced risk of schizophrenia. NeuroImage. 2006 Jul 15;31(4):1850–8.

137. Lee J, Cohen MS, Engel SA, Glahn D, Nuechterlein KH, Wynn JK, et al. Regional brain activity during early visual perception in unaffected siblings of schizophrenia patients. Biol Psychiatry. 2010 Jul 1;68(1):78–85.

138. Stevens JS, Jovanovic T, Fani N, Ely TD, Glover EM, Bradley B, et al. Disrupted amygdala-prefrontal functional connectivity in civilian women with posttraumatic stress disorder. J Psychiatr Res. 2013 Oct;47(10):1469–78.

139. Simmons AN, Matthews SC, Strigo IA, Baker DG, Donovan HK, Motezadi A, et al. Altered amygdala activation during face processing in Iraqi and Afghanistani war veterans. Biol Mood Anxiety Disord. 2011 Oct 12;1:6.

140. Shin LM, McNally RJ, Kosslyn SM, Thompson WL, Rauch SL, Alpert NM, et al. Regional Cerebral Blood Flow During Script-Driven Imagery in Childhood Sexual Abuse-Related PTSD: A PET Investigation. AJP. 1999 Apr;156(4):575–84.

141. Phan KL, Britton JC, Taylor SF, Fig LM, Liberzon I. Corticolimbic Blood Flow During Nontraumatic Emotional Processing in Posttraumatic Stress Disorder. Archives of General Psychiatry. 2006 Feb 1;63(2):184–92.

142. Nawijn L, van Zuiden M, Koch SBJ, Frijling JL, Veltman DJ, Olff M. Intranasal oxytocin increases neural responses to social reward in post-traumatic stress disorder. Soc Cogn Affect Neurosci. 2016 Oct 25;12(2):212–23.

143. Lanius RA, Williamson PC, Densmore M, Boksman K, Gupta MA, Neufeld R w., et al. Neural Correlates of Traumatic Memories in Posttraumatic Stress Disorder: A Functional MRI Investigation. AJP. 2001 Nov;158(11):1920–2.

144. Tognin S, Pettersson-Yeo W, Valli I, Hutton C, Woolley J, Allen P, et al. Using Structural Neuroimaging to Make Quantitative Predictions of Symptom Progression in Individuals at Ultra-High Risk for Psychosis. Frontiers in Psychiatry [Internet]. 2014 [cited 2022 Jun 22];4. Available from: https://www.frontiersin.org/article/10.3389/fpsyt.2013.00187

145. Modinos G, Şimşek F, Azis M, Bossong M, Bonoldi I, Samson C, et al. Prefrontal GABA levels, hippocampal resting perfusion and the risk of psychosis. Neuropsychopharmacol. 2018 Dec;43(13):2652–9.

146. Krakauer K, Nordentoft M, Glenthøj BY, Raghava JM, Nordholm D, Randers L, et al. White matter maturation during 12 months in individuals at ultra-high-risk for psychosis. Acta Psychiatrica Scandinavica. 2018;137(1):65–78.

147. Kindler J, Schultze-Lutter F, Hauf M, Dierks T, Federspiel A, Walther S, et al. Increased Striatal and Reduced Prefrontal Cerebral Blood Flow in Clinical High Risk for Psychosis. Schizophrenia Bulletin. 2018 Jan 13;44(1):182–92.

148. Cropley VL, Lin A, Nelson B, Reniers RLEP, Yung AR, Bartholomeusz CF, et al. Baseline grey matter volume of non-transitioned “ultra high risk” for psychosis individuals with and without attenuated psychotic symptoms at long-term follow-up. Schizophrenia Research. 2016 Jun 1;173(3):152–8.

149. Peterson BS, Wang Z, Horga G, Warner V, Rutherford B, Klahr KW, et al. Discriminating Risk and Resilience Endophenotypes From Lifetime Illness Effects in Familial Major Depressive Disorder. JAMA Psychiatry. 2014 Feb;71(2):136–48.

150. Liu CH, Ma X, Wu X, Fan TT, Zhang Y, Zhou FC, et al. Resting-state brain activity in major depressive disorder patients and their siblings. Journal of Affective Disorders. 2013 Jul 1;149(1):299–306.

151. Van Rooij S, Rademaker A, Kennis M, Vink M, Kahn R, Geuze E. Neural correlates of trauma-unrelated emotional processing in war veterans with PTSD. Psychological medicine. 2014 Jul 18;1–13.

152. Deveney CM, Connolly ME, Jenkins SE, Kim P, Fromm SJ, Brotman MA, et al. Striatal Dysfunction During Failed Motor Inhibition in Children at Risk for Bipolar Disorder. Prog Neuropsychopharmacol Biol Psychiatry. 2012 Aug 7;38(2):127–33.

153. Admon R, Lubin G, Stern O, Rosenberg K, Sela L, Ben-Ami H, et al. Human vulnerability to stress depends on amygdala's predisposition and hippocampal plasticity. Proc Natl Acad Sci U S A. 2009 Aug 18;106(33):14120–5.

154. Sekiguchi A, Sugiura M, Taki Y, Kotozaki Y, Nouchi R, Takeuchi H, et al. Brain structural changes as vulnerability factors and acquired signs of post-earthquake stress. Mol Psychiatry. 2013 May;18(5):618–23.
