# Supplementary material for: Neuroimaging correlates of psychological resilience: an Open Science systematic review and meta-analysis
Source: Front Neuroimaging. 2025 May 13;4:1487888. doi: 10.3389/fnimg.2025.1487888 (PMC12106531; doi:10.3389/fnimg.2025.1487888)
Supplement: Supplementary file 4 [file Data_Sheet_4.zip › Table S1 Resilience Meta-analyses by Modality.docx]

| **Table S1. Brain regions associated with psychological resilience by neuroimaging modality** | | | | | | | |
| --- | --- | --- | --- | --- | --- | --- | --- |
| **Task-based functional MRI (k=65)** | | | | | | | |
| Cluster # | x | y | z | ALE | P | Z | Label (Nearest Gray Matter within 5mm) |
| 1 | 22 | -6 | -16 | 0.0449 | 2.31E-13 | 7.24 | Right Cerebrum.Limbic Lobe.Parahippocampal Gyrus.Gray Matter.Amygdala |
| 2 | -24 | -4 | -18 | 0.0242 | 5.41E-07 | 4.88 | Left Cerebrum.Limbic Lobe.Parahippocampal Gyrus.Gray Matter.Amygdala |
| 3 | 34 | 26 | -8 | 0.0334 | 1.03E-09 | 5.99 | Right Cerebrum.Sub-lobar.Insula.Gray Matter.Brodmann area 13 |
| **Resting-state functional MRI (k=34)** | | | | | | | |
| Cluster # | x | y | z | ALE | P | Z | Label (Nearest Gray Matter within 5mm) |
| 1 | -24 | -6 | -18 | 0.0225 | 7.34E-07 | 4.82 | Left Cerebrum.Limbic Lobe.Parahippocampal Gyrus.Gray Matter.Amygdala |
| 2 | 20 | -6 | -16 | 0.0227 | 6.19E-07 | 4.85 | Right Cerebrum.Limbic Lobe.Parahippocampal Gyrus.Gray Matter.Amygdala |
| 3 | -32 | 24 | 48 | 0.0265 | 4.34E-08 | 5.35 | Left Cerebrum.Frontal Lobe.Middle Frontal Gyrus.Gray Matter.Brodmann area 8 |
| **Structural MRI (k=34)** | | | | | | | |
| Cluster # | x | y | z | ALE | P | Z | Label (Nearest Gray Matter within 5mm) |
| 1 | 4 | 42 | 16 | 0.0245 | 8.13E-08 | 5.24 | Right Cerebrum.Limbic Lobe.Anterior Cingulate.Gray Matter.Brodmann area 32 |
| 1 | 2 | 44 | 12 | 0.0234 | 1.81E-07 | 5.09 | Left Cerebrum.Limbic Lobe.Anterior Cingulate.Gray Matter.Brodmann area 32 |
| 2 | -20 | 6 | -26 | 0.0140 | 1.10E-04 | 3.69 | Left Cerebrum.Limbic Lobe.Uncus.Gray Matter.Brodmann area 34 |
| 3 | -38 | 8 | -4 | 0.0169 | 1.74E-05 | 4.14 | Left Cerebrum.Sub-lobar.Claustrum.Gray Matter.* |
