# Supplementary material for: Neuroimaging correlates of psychological resilience: an Open Science systematic review and meta-analysis
Source: Front Neuroimaging. 2025 May 13;4:1487888. doi: 10.3389/fnimg.2025.1487888 (PMC12106531; doi:10.3389/fnimg.2025.1487888)
Supplement: Supplementary file 4 [file Data_Sheet_4.zip › Table S1 Resilience Meta-analyses by Modality.pdf]

Table S1. Brain regions associated with psychological resilience by neuroimaging modality							
**Task-based functional MRI (k=65)**							
Cluster #	x	y	z	ALE	P	Z	Label (Nearest Gray Matter within 5mm)
1	22	-6	-16	0.0449	2.31E-13	7.24	Right Cerebrum.Limbic Lobe.Parahippocampal Gyrus.Gray Matter.Amygdala
2	-24	-4	-18	0.0242	5.41E-07	4.88	Left Cerebrum.Limbic Lobe.Parahippocampal Gyrus.Gray Matter.Amygdala
3	34	26	-8	0.0334	1.03E-09	5.99	Right Cerebrum.Sub-lobar.Insula.Gray Matter.Brodmann area 13
**Resting-state functional MRI (k=34)**							
Cluster #	x	y	z	ALE	P	Z	Label (Nearest Gray Matter within 5mm)
1	-24	-6	-18	0.0225	7.34E-07	4.82	Left Cerebrum.Limbic Lobe.Parahippocampal Gyrus.Gray Matter.Amygdala
2	20	-6	-16	0.0227	6.19E-07	4.85	Right Cerebrum.Limbic Lobe.Parahippocampal Gyrus.Gray Matter.Amygdala
3	-32	24	48	0.0265	4.34E-08	5.35	Left Cerebrum.Frontal Lobe.Middle Frontal Gyrus.Gray Matter.Brodmann area 8
**Structural MRI (k=34)**							
Cluster #	x	y	z	ALE	P	Z	Label (Nearest Gray Matter within 5mm)
1	4	42	16	0.0245	8.13E-08	5.24	Right Cerebrum.Limbic Lobe.Anterior Cingulate.Gray Matter.Brodmann area 32
1	2	44	12	0.0234	1.81E-07	5.09	Left Cerebrum.Limbic Lobe.Anterior Cingulate.Gray Matter.Brodmann area 32
2	-20	6	-26	0.0140	1.10E-04	3.69	Left Cerebrum.Limbic Lobe.Uncus.Gray Matter.Brodmann area 34
3	-38	8	-4	0.0169	1.74E-05	4.14	Left Cerebrum.Sub-lobar.Claustrum.Gray Matter.*
