# Supplementary material for: Neuroimaging correlates of psychological resilience: an Open Science systematic review and meta-analysis
Source: Front Neuroimaging. 2025 May 13;4:1487888. doi: 10.3389/fnimg.2025.1487888 (PMC12106531; doi:10.3389/fnimg.2025.1487888)
Supplement: Supplementary file 5 [file Data_Sheet_5.pdf]

Description of Supplementary materials

Data Sheet 1 includes full curated zotero library for the systematic review, as well as the bibliography for the 154 articles included in the meta-analyses.

Data Sheet 2 is the GingerALE meta-analysis described in Figure 2.

Data Sheet 3 is the GingerALE meta-analyses by disorder (bipolar disorder BD, major depressive disorder MDD, post-traumatic stress disorder PTSD, and schizophrenia SZ) and contrasts between disorders.

Data Sheet 4 is the GingerALE meta-analyses by MRI modality (resting-state fMRI or rs-fMRI, task-based fMRI or t-fMRI, and structural MRI or sMRI).
